# Synergistic doping with Ag, CdO, and ZnO to overcome electron-hole recombination in TiO_2_ photocatalysis for effective water photo splitting reaction

**DOI:** 10.3389/fchem.2023.1301172

**Published:** 2023-11-06

**Authors:** Nehal A. Erfan, Mohamed S. Mahmoud, Hak Yong Kim, Nasser A. M. Barakat

**Affiliations:** ^1^ Chemical Engineering Department, Minia University, El-Minia, Egypt; ^2^ Department of Engineering, University of Technology and Applied Sciences, Suhar, Oman; ^3^ Department of Nano Convergence Engineering, Jeonbuk National University, Jeonju, Republic of Korea; ^4^ Department of Organic Materials and Fiber Engineering, Jeonbuk National University, Jeonju, Republic of Korea

**Keywords:** heterojunction, photocatalyst, water photo splitting, solar irradiation, titanium oxide doping

## Abstract

This manuscript is dedicated to a comprehensive exploration of the multifaceted challenge of fast electron-hole recombination in titanium dioxide photocatalysis, with a primary focus on its critical role in advancing the field of water photo splitting. To address this challenge, three prominent approaches—Schottky barriers, Z-scheme systems, and type II heterojunctions—were rigorously investigated for their potential to ameliorate TiO_2_’s photocatalytic performance toward water photo splitting. Three distinct dopants—silver, cadmium oxide, and zinc oxide—were strategically employed. This research also delved into the dynamic interplay between these dopants, analyzing the synergetic effects that arise from binary and tertiary doping configurations. The results concluded that incorporation of Ag, CdO, and ZnO dopants effectively countered the fast electron-hole recombination problem in TiO_2_ NPs. Ag emerged as a critical contributor at higher temperatures, significantly enhancing photocatalytic performance. The photocatalytic system exhibited a departure from Arrhenius behavior, with an optimal temperature of 40°C. Binary doping systems, particularly those combining CdO and ZnO, demonstrated exceptional photocatalytic activity at lower temperatures. However, the ternary doping configuration involving Ag, CdO, and ZnO proved to be the most promising, surpassing many functional materials. In sum, this study offers valuable insights into how Schottky barriers, Z-scheme systems, and type II heterojunctions, in conjunction with specific dopants, can overcome the electron-hole recombination challenge in TiO_2_-based photocatalysis. The results underscore the potential of the proposed ternary doping system to revolutionize photocatalytic water splitting for efficient green hydrogen production, significantly advancing the field’s understanding and potential for sustainable energy applications.

## 1 Introduction

The urgent need for sustainable and environmentally friendly approaches to produce green hydrogen as a replacement for fossil fuels has spurred extensive research in the field of photocatalysis (Akhtar et al., 2017; Fazal et al., 2023; Zafar et al., 2023). Photocatalytic water splitting using titanium dioxide (TiO_2_) has emerged as a promising route to harness solar energy for hydrogen generation. Photocatalysis using titanium dioxide represents an environmentally friendly and promising technology for the water splitting process, a crucial step in renewable hydrogen production and tackling global energy challenges. TiO_2_ offers several distinct advantages in this context. Firstly, it is abundant and cost-effective, making it well-suited for large-scale applications. TiO_2_ is known for its photostability and chemical inertness under UV light, ensuring long-term performance without corrosion concerns. Moreover, its bandgap structure matches the solar spectrum, enabling efficient utilization of natural sunlight and reducing energy costs. Importantly, TiO_2_ is environmentally benign and non-toxic, aligning with sustainability objectives. Its efficient charge separation properties, tunability through doping, and scalability make it a versatile candidate for various photocatalytic applications. Additionally, TiO_2_ photocatalysis is characterized by dual-functionality, allowing both hydrogen production and water purification. With its longevity and potential for hybrid systems by combining with other materials, such as noble metals or semiconductors, TiO_2_ photocatalysis contributes to greener and more sustainable solutions for renewable hydrogen production and water treatment, promising a cleaner and more environmentally conscious future (Barakat et al., 2014; Eidsvåg et al., 2021).

However, a formidable challenge hindering the efficient use of TiO_2_-based photocatalysts is the rapid recombination of photogenerated electron-hole pairs (e^−^/h^+^), significantly impeding the overall hydrogen production rate.

Several innovative strategies have been proposed to address this electron-hole recombination issue, each offering unique advantages:

1. Semiconductor heterojunctions: One promising strategy involves coupling TiO_2_ with semiconductors to form heterojunctions. This approach creates diverse junction types (type I, type II, and type III) based on the properties of the incorporated semiconductor materials. Oxides such as ZnO, WO_3_, SnO_2_, and Fe_3_O_4_ have been explored for their potential to reduce recombination. In these heterojunctions, the lower-gap energy semiconductor sensitizes TiO_2_ by initiating the excitation process, facilitating efficient charge separation, and suppressing recombination (Ahmad et al., 2019; Bera et al., 2019; Zhang et al., 2020; Chakhtouna et al., 2021).
2. Z-Scheme photocatalytic systems: Another effective approach is the creation of Z-scheme photocatalytic systems, where charge carrier recombination is efficiently suppressed. This is achieved by combining photogenerated electrons in the conduction band (CB) of one semiconductor with holes in the valence band (VB) of another semiconductor. This results in remaining electrons in the CB and holes in the VB, both possessing robust redox capabilities, leading to superior photocatalytic activity. Z-scheme heterojunctions, such as ZnS/SnS_2_, CeO_2_/BiOBr, and MoO_3_/Bi_2_O_4_, have demonstrated exceptional performance in applications like antibiotic removal from wastewater (Jiang et al., 2020; Xia et al., 2020; Jia et al., 2021; Xie et al., 2023).
3. Noble metal nanoparticles incorporation: The incorporation of noble metal nanoparticles (e.g., Ag, Au, Pt, and Pd) onto the TiO_2_ surface has been explored to enhance photocatalytic activity. These noble metals act as electron traps by forming Schottky barriers at the TiO_2_-metal junctions. This promotes efficient interfacial charge transfer and delays the recombination of electron-hole pairs, leading to improved photocatalytic performance (Harikishore et al., 2014; Endo et al., 2018; Yaqoob et al., 2020; Bilal et al., 2022).
4. Non-metal and transition ions doping: Doping TiO_2_ with non-metals (p-block elements) such as nitrogen, carbon, and sulfur enhances its responsiveness to visible light and mitigates recombination by modifying its electronic band structure. Nitrogen, with its small ionization energy and atomic size similar to oxygen, remains a commonly used dopant. Transition metals, possessing unfilled d-electron structures, can transfer electrons from their 3d levels to TiO_2_’s conduction band, accommodating more electrons and effectively trapping photogenerated carriers, thus reducing recombination (Motlak et al., 2014; Yadav and Jaiswar, 2017; Barakat et al., 2018; Žener et al., 2018).
5. Graphene and derivatives modification: The incorporation of graphene and its derivatives has gained attention due to their unique structure, high electron mobility, and band level modification capabilities. These materials prevent charge recombination by modifying the valence and conduction band levels of TiO_2_ (Gunti et al., 2015; Mohamed et al., 2017; Kim et al., 2019; Khalil et al., 2020).
6. Sensitization with dyes: Sensitizing TiO_2_ with dyes has been explored to enhance its photocatalytic activity. However, challenges related to the chemical and thermal stability of these dyes have been encountered (Islam et al., 2001; Li et al., 2019; Barakat et al., 2022).

Despite extensive research on these approaches for wastewater treatment, limited attention has been given to comparing their performance in water photo splitting reactions. It is worth mentioning that, among the aforementioned approaches, the first three showed the best efficacy and the most widely used.

Zinc oxide (ZnO) possesses a band gap energy closely aligned with that of TiO_2_, measuring 3.37 eV and 3.2 eV, respectively. However, notable disparities exist in the conduction and valence band energies of these two oxides. Specifically, in numerical terms, the conduction bands are situated at −0.25 eV for TiO_2_ and −0.6 eV for ZnO, while the corresponding valence bands are found at 2.95 eV for TiO_2_ and 2.77 eV for ZnO (Burnside et al., 1999; Abbas et al., 2018; Menon et al., 2019). Consequently, it is reasonable to assert that the integration of TiO_2_ with ZnO results in the formation of a type II heterojunction (Shen et al., 2014). In contrast, Cadmium oxide (CdO), characterized as an *n*-type metallic oxide, exhibits a shorter direct band gap of 2.3 eV, which aligns well with the energy of visible light photons. Furthermore, considering its notably higher conduction band position compared to TiO_2_ (0.74 eV), combining CdO with TiO_2_ has the potential to establish a Z-scheme photocatalytic system.

This study focuses on enhancing the photocatalytic activity of titanium dioxide nanoparticles by incorporating three different, in nature, dopants: ZnO, CdO, and Ag. These dopants lead to the formation of three distinct photocatalytic systems: type II heterojunction, Z-scheme, and the formation of a Schottky barrier with noble metals, respectively. The investigation also explores the influence of varying dopant concentrations individually and in binary and ternary combinations, revealing the synergistic effects on hydrogen production from water splitting under visible light radiation. This research is of paramount importance as it sheds light on the distinct effects of these photocatalytic systems and their synergistic contributions to improving the efficiency of green hydrogen production. Furthermore, in this work, the kinetics of the water splitting reactions have been investigated, particularly, the dependance of the gross reactions on the temperature. The results confirmed that the reaction process involves both the photocatalysts and co-catalyst contribution to the value of the activation.

## 2 Materials and methods

### 2.1 Materials

The chemicals consumed in this study have been used without further modifications. Titanium (IV) isopropoxide (Ti(OCH(CH_3_)_2_)_4_) and polyvinyl pyrolidone (PVP, Mw = 130,000), purchased from Merck, were used for synthesis of TiO_2_ nanoparticles. Silver nitrate (AgNO_3_ assay 99.9%), zinc acetate dihydrates (Zn(CH₃COO)₂·2H₂O, assay 99.0%), and cadmium acetate dihydrate (Cd(CH₃COO)₂·2H₂O, assay 99.0%) were purchased from Showa Co. Japan. They have been added as a precursor of noble and transition metals admission into titania nanoparticles within the doping step. The solvent used in this study was ethanol (CH_3_CH_2_OH, analytical-grade, assay 99.8%–100%, Alpha chemicals, USA). For the preparation step, acetic acid (CH_3_COOH, assay 99%, Alpha chemicals, USA), sodium sulfide (Na_2_SO_3_, Assay 98%, Merck), and sodium sulfate (Na_2_SO_4_, assay 55%–60%, Merck) have been used.

### 2.2 Fabrication of TiO_2_ and Ag/Cd/Zn-doped TiO_2_ nanoparticles

The sol-gel process is a method for producing solid materials, such as metal oxides, in the form of micro and nano-sized particles. This is achieved through a series of chemical and physical steps, beginning with the dissolution of dopant and host precursors in solvents to initiate the reaction. After doping, the remaining liquid solvent is removed through a drying process, which results in shrinkage and densification. The rate at which the solvent is removed is determined by the porosity distribution within the gel. The final microstructure of the material is strongly influenced by any changes made during the synthesis process. To enhance the structural stability and mechanical properties of the material, thermal treatment is necessary. This involves processes such as polycondensation, sintering, densification, and grain growth. Pure TiO_2_ nanoparticles were first prepared as a reference using the sol-gel technique (Sang et al., 2014). To form a final polymer solution of 25 wt. %, 3 g of PVP polymer granules were dissolved in ethanol and stirred at 30°C to complete the mixture solubility. An equal amount of ethanol and acetic acid was mixed for 5 min. Then, 2 mL of titanium isopropoxide was added with further stirring for 15 min. Afterward, the second mixture was added to the polymer solution with continuous stirring to obtain transparent sol-gel. The sol-gel solution was then dried at 80°C for 24 h. Lastly, calcination of the resultant sample is done at 700°C for 5 h in an inert N_2_ atmosphere.

For the metal-doped TiO_2_ nanoparticles, the procedure was similarly performed after adding specific amounts from AgNO_3_, Zn(CH₃COO)₂·2H₂O, and Cd(CH₃COO)₂·2H₂O dissolved in 2 mL ethanol to the PVP- Ti(OCH(CH_3_)_2_)_4_ transparent solution before drying. Different samples of single TiO_2_ doping were prepared by changing the amount of salt to produce nanoparticles having 0.5, 1, and 2 wt. % dopants compared with pristine TiO_2_ nanoparticles. The final solution was dried for 24 h at 80°C. And the solid product was exposed to calcination at 700°C for 5 h in an inert N_2_ atmosphere. For binary and ternary co-doping, specific amounts of metallic precursors have been dissolved in 2 mL ethanol and mixed with PVP- Ti(OCH(CH_3_)_2_)_4_ solution using the same aforementioned procedure.

### 2.3 Physical, chemical, and optical characterization

The characterizations have been performed in the Central lab for Microanalysis and Nanotechnology, Minia University. The morphology and the structure of the surfaces for the as-prepared nanoparticles was captured by the transmission electron microscope (TEM, JEOL JEM-2200FS, Japan). It runs at 200 kV and is equipped with EDX. A Rigaku X-ray diffractometer (XRD, Rigaku Co., Japan) with Cu Kα (*λ* = 1.54056 Å) radiation over a 2θ range from 10° to 80° was used for the phase and crystallinity characterization of the prepared nanomaterials. The optical properties were investigated using HP 8453 UV-visible spectroscopy system, Germany.

### 2.4 Experiment for photons–induced water splitting

**FIGURE 1:**
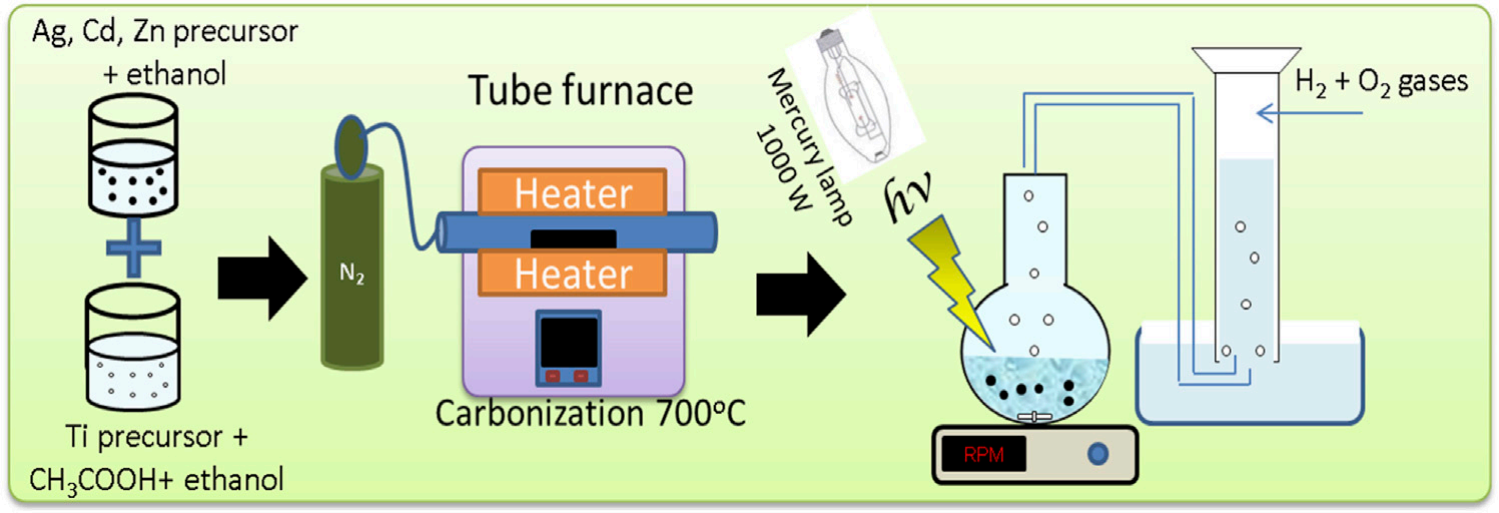
Schematic diagram of the set used for water splitting.

A 1000 W mercury lamp was used in the experiments for evaluating the photocatalytic activity of the prepared nanoparticles in water (Wetchakun et al., 2019). Initially, a solution of 0.1 g photocatalyst was added to 100 mL of 0.5 M Na_2_S/Na_2_SO_3_ sacrificial agent. The suspension was placed in a well-sealed round-bottom flask, where a rubber tube exits from one opening in the bottom flask. The evolved gases were collected by water displacing in a water-filled inverted graduated cylinder where a rubber tube was immersed (as shown in Figure 1). The round-bottom flask was covered by a temperature-controlled water bath for controlling the reaction temperature. H_2_ and O_2_ with a molar ratio of 2:1 were the constituents of the evolved gas from the photocatalytic water-splitting process. The accumulated hydrogen volume was calculated by following the change of volume above the water level using the following equation:

$$n=\frac{2\times 273\times V}{3\times 22.4\times m\times T}$$ (1)

where n is the number of moles of H_2_ [mmol/g], m is the mass of the photocatalyst [g], V is the volume of the gas [mL], and T is the temperature of the solution [K].

## 3 Results and discussions

### 3.1 Catalyst characterization

**FIGURE 2:**
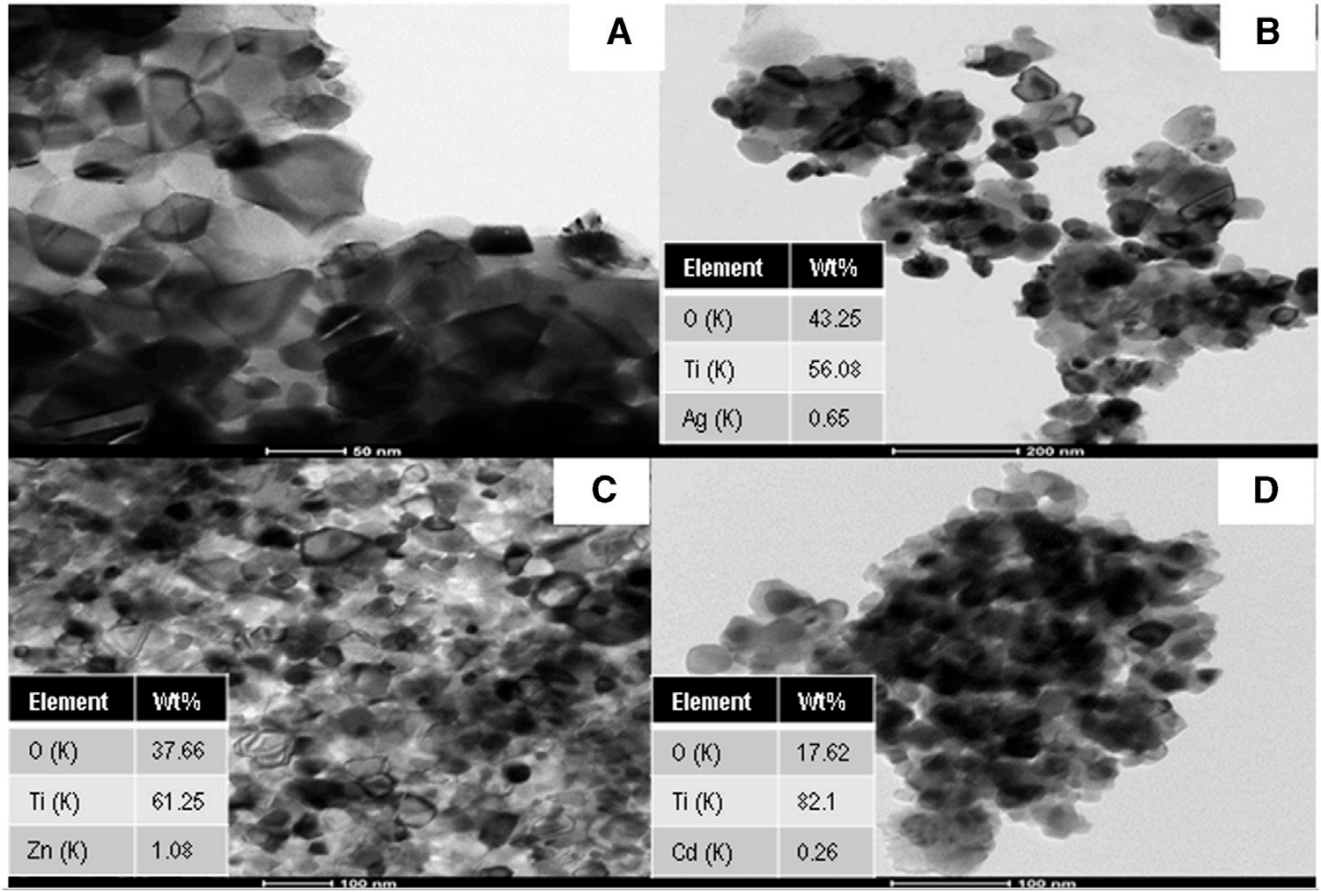
TEM images and corresponding EDX results for **(A)** TiO_2_ nanoparticles, **(B)** Ag-doped TiO_2_ nanoparticles, **(C)** Zn-doped TiO_2_ nanoparticles, and **(D)** Cd-doped TiO_2_ nanoparticles.

Figure 2 displays the TEM images of Ag, Zn, and Cd-doped TiO_2_ nanoparticles compared to pristine TiO_2_ nanoparticles. There are black dots with different crystal structure than the main matrix indicating the presence of Ag, ZnO, and CdO nanoparticles within the TiO_2_ matrix. Actually, because of the significant difference in the crystal parameters between Ag, ZnO, CdO and TiO_2_, their mixture cannot form a solid solution. Therefore, the final structure is Ag, ZnO, and CdO-doped TiO_2_ nanoparticles. It is noteworthy mentioning that TEM results confirm that the introduced nanoparticles average size is less than 100 nm. Furthermore, it can be noticed from Figure 2 that the dopants have good distribution within the TiO_2_ matrix, which results in good photocatalytic activities of the introduced nanoparticles. In addition, the EDX analysis has been used to confirm the presence of atoms in the host TiO_2_ nanoparticles. It is also noticeable that the wt.% of Ag, Zn, and Cd were found to be 0.65, 1.08, and 0.26 wt.%, respectively (Figures 2B–D). It is worth mentioning that the binary and ternary co-doping formulations reveals almost similar TEM images (data are not shown).

**FIGURE 3:**
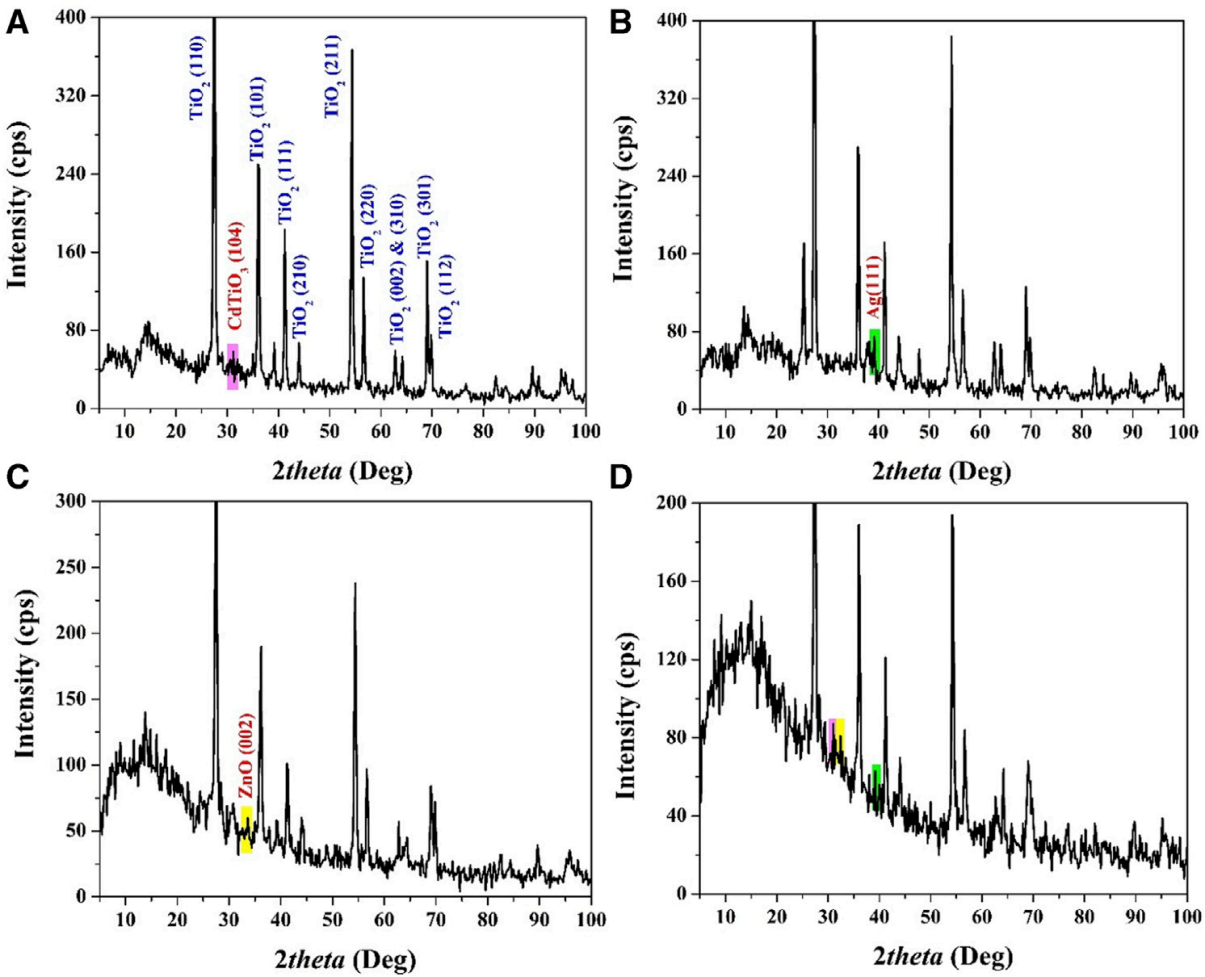
XRD patterns for the prepared CdO-doped TiO_2_; **(A)**, Ag-doped TiO_2_; **(B)**, ZnO-doped TiO_2_; **(C)**, and CdO&ZnO&Ag -doped TiO_2_; **(D)** nanoparticles.

**FIGURE 4:**
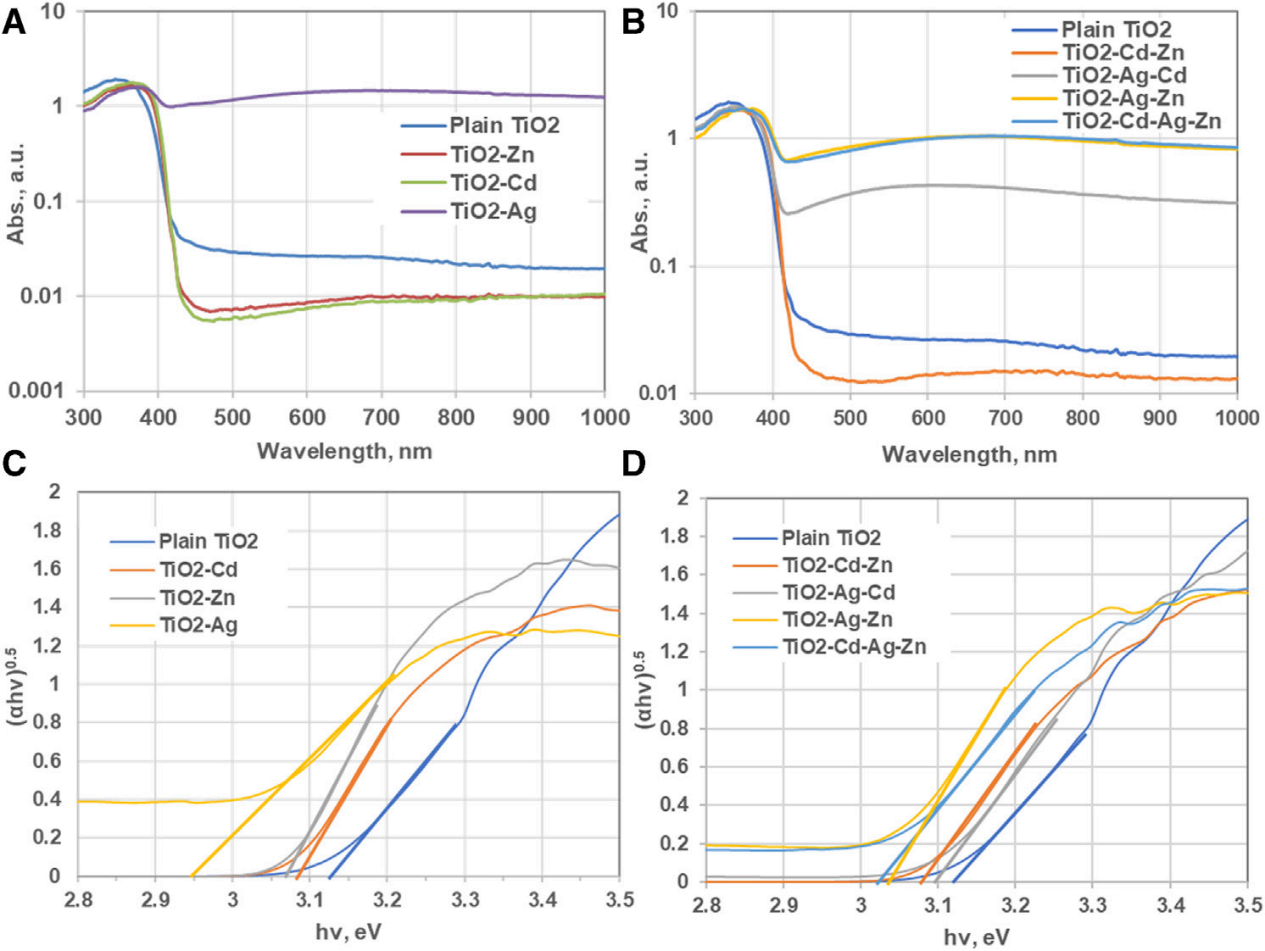
**(A, C)** Absorbance versus wavelength curve of TiO_2_ nanoparticles compared to different dopants. **(B, D)** Allowed direct energy band gap of plain TiO_2_ nanoparticle and that for TiO_2_ prepared with various dopants.

X-ray Diffraction (XRD) is a pivotal and dependable characterization technique in materials science, offering valuable insights into the structural composition of crystalline materials. In this study, XRD analysis played a central role in confirming the formation of specific phases and the incorporation of dopants within the synthesized nanoparticles. The results from Figures 3A–C, 4 underscore the significance of XRD in validating these critical aspects of the materials.

The XRD pattern in Figure 3A provides compelling evidence of the synthesized nanoparticles’ crystal structure. The emergence of distinct peaks at specific 2θ values (27.4°, 36°, 39.2°, 41.2°, 44°, 54.3°, 56.6°, and 64.0°) corresponds to the reflections from various crystallographic planes (e.g., (110), (101), (200), (111), (210), (211), and (220)) of tetragonal TiO2 (rutile). This unequivocally confirms the formation of the rutile phase of TiO2 in the synthesized nanoparticles.

Additionally, the appearance of a peak at 2θ = 31.15°, attributed to the (104) crystal plane, suggests the formation of CdTiO_3_ solid solution. This formation is due to the decomposition of cadmium acetate and its subsequent incorporation into the TiO_2_ crystal lattice. The small peak intensity indicates that the CdO content in the nanoparticles is relatively low, accounting for only 2 wt%. The XRD pattern in Figure 3B reveals the successful incorporation of silver (Ag) into the TiO_2_ nanoparticles. Alongside the rutile phase peaks, a distinct peak at 2θ = 38.12° corresponding to the (111) crystal plane is evident. This peak aligns precisely with the JCDPS database reference for silver (Ag). The presence of this peak confirms the successful doping of TiO_2_ with silver nanoparticles.

Figure 3C illustrates the XRD pattern of nanoparticles containing zinc oxide (ZnO). Notably, in addition to the rutile phase peaks, a conspicuous peak appears at 2θ = 33.2°, signifying the presence of ZnO. This peak corresponds to the (002) crystal plane of zinc oxide, corroborating the successful incorporation of ZnO into the TiO2 nanoparticles. Figure 4D showcases the XRD pattern of nanoparticles incorporating all three dopants. The pattern unequivocally confirms the presence of the dopant nanoparticles. While the specific peak positions may vary due to the different crystal structures of the dopant materials, the appearance of representative peaks validates the successful integration of multiple dopants into the TiO_2_ nanoparticles.

Overall, X-ray Diffraction (XRD) has proven to be an indispensable tool in characterizing the synthesized nanoparticles and validating the incorporation of specific dopants. These results provide a solid foundation for further investigations into the photocatalytic properties of these modified TiO_2_ nanoparticles, as discussed in subsequent sections.

One of the ways to improve the efficiency of photocatalytic reactions of pristine TiO_2_ nanoparticles is to modify the electronic structure, optical properties, and charge transfer dynamics of the nanoparticles, leading to enhanced photocatalytic activity. To understand how the dopants affect the performance of the TiO_2_ nanoparticles, it is important to investigate how their photo-characteristics differ from those of the pristine ones. This can be done by measuring the absorption spectra and photoluminescence spectra of the doped and undoped nanoparticles under different wavelengths and intensities of light. From these spectra, one can gain insights into the band gap, defect states, charge separation and recombination processes of the nanoparticles, and how they are influenced by the dopants. Figures 4A, C shows the UV-vis absorption spectra of the undoped and doped TiO_2_ nanoparticles. The fundamental absorption edge of TiO_2_ nanoparticles was observed at 420 nm which is slightly higher than the typical fundamental absorption edge of TiO_2_ nanoparticles, which is in the ultraviolet range, around 362 nm for pure TiO_2_ (Zheng et al., 2021). But it can be shifted to the visible range by introducing impurities, disorder, hydrogenation, annealing, or metal doping. This shift was reported by Muniyappan et al. (2017) and Tesfaye Jule et al. (2021) for TiO_2_ anatase nanoparticles can be observed at 385 nm, which corresponds to the band gap of 3.2 eV. It is noticeable from Figure 4A that for single doping of TiO_2_ nanoparticles, only Ag doping enhanced the percentage of visible light absorption, which is same as reported in (Yildirim, 2021). Whereas doping with ZnO and CdO did not enhance the photons absorption. Regarding Cd doping, it possessed a lower percentage of absorbance because CdO acts as transparent conducting transition metal oxide (TCO). The same finding is also observed for ZnO doping as ZnO-doped TiO_2_ nanoparticles revealed relatively higher transmittance %. This high transmittance favored the use of ZnO/TiO_2_ thin films as a photodetector and solar detector applications (Serkan et al., 2021). Regarding bi and tri metallic doping (Figure 4C), the adverse effect of bi and tri doping can be noticed. Doping TiO_2_ with both Cd and Zn decreased the absorption of visible light photons for the reasons explained earlier, while it increases by doping with other bi and tri metallic combinations.

The direct and indirect band gap energy (eV) of the prepared samples can be calculated by Tauc method using the following equation (Mahmoud et al., 2018):

$${\left(\alpha hv\right)}^{1/\gamma }=B\left(hv-E_{g}\right)$$ (2)

Where *α* is the absorption coefficient, *h* is the Planck constant, *ν* is the photon frequency (h*v* = 1240/wavelength), *B* is the proportionality constant, *E*_*g*_ is the band gap energy, and γ is a constant. The value of γ varies according to direct and indirect band gaps; it takes a value of γ = 2 for indirect allowed and γ = 1/2 for the direct allowed absorption according to the optical transition properties of the catalyst. Figures 4B,D shows the Tauc plot of photons energy at direct band gap energy as function of (*αhυ*)^2^. The band gap energy is obtained by extending the slopes of the linear part of the curves to the x-axis.

The values of the band gap energies of pure, and M-doped TiO_2_ are shown in Table 1.

**TABLE 1:** Indirect bandgap energy for plain TiO_2_ nanoparticles and different dopants.

Nanoparticle	Indirect bandgap energy, eV
TiO_2_	3.13
Cd-doped TiO_2_	3.08
Zn-doped TiO_2_	3.07
Ag-doped TiO_2_	2.94
Cd/Zn-doped TiO_2_	3.08
Ag/Cd-doped TiO_2_	3.1
Ag/Zn-doped TiO_2_	3.04
Cd/Ag/Zn-doped TiO_2_	3.02

The bandgap energy for the prepared pristine TiO_2_ nanoparticles was 3.13 eV due to temperature effect or presence of impurities and defects. The most pronounced decrease in the bandgap energy was achieved by Ag doping while other single, bi, and tri doping reduced the bandgap slightly with insignificant shift to the red region (Pérez-Larios et al., 2012; Luévano-Hipólito and Torres-Martínez, 2017). For Ag-doped TiO_2_ nanoparticles, Ag nanoparticles have a strong surface plasmon resonance (SPR) property. The plasmonic effect of Ag nanoparticles compared to other noble metals facilitates the enhancement of the optical properties of the host TiO_2_ photocatalyst. Therefore, Ag-doped TiO_2_ photocatalyst can absorb light photons in the UV and visible regions, which increases the efficiency of photocatalytic reactions (Yildirim, 2021). While for doping with ZnO, it is reported that ZnO nanoparticles are semiconductors and have a wide bandgap (Luévano-Hipólito and Torres-Martínez, 2017; Yildirim, 2021), therefore, doping TiO_2_ with ZnO was expected not to alter the bandgap of the combination. Similarly, a possible reason for the increased hydrogen production rate is that the CdO-doped TiO_2_ nanoparticles allowed more visible light radiation to pass through and be trapped by the TiO_2_ nanoparticles’ lattice. This improved the absorption of photons and compensated for the lack of band gap reduction. Moreover, the CdO-rich sites probably prevented the electron-hole pair from recombining too quickly. This is consistent with what Luminita Andronic et al. (Andronic et al., 2009) reported about the effect of Cd doping on TiO_2_ films. They stated that a certain amount of Cd could slow down the recombination rate of the electron/hole pair.

### 3.2 Photocatalytic water splitting experiments

In the context of water photo splitting reactions, scavengers play a crucial role in improving the efficiency and selectivity of the process. While the concept is promising for sustainable hydrogen production, several challenges, including the rapid recombination of photo-generated electron-hole pairs (e^−^/h^+^), can hinder the overall efficiency (Barakat et al., 2023). Scavengers are substances introduced into the reaction system to address these challenges and enhance the photocatalytic process. Scavengers can assist in the separation of electrons and holes, preventing them from recombining prematurely. By capturing one of the charge carriers (either electrons or holes), scavengers extend the lifetime of the remaining charge carrier, allowing it to engage in photocatalytic reactions. This separation enhances the chances of electrons and holes reaching the catalyst surface to drive the desired redox reactions. Scavengers can be designed to selectively capture either electrons or holes, depending on the specific needs of the photocatalytic system (Wang et al., 2023). For instance, electron scavengers, such as sacrificial agents, capture and utilize excess electrons to reduce water to hydrogen. Conversely, hole scavengers, like sacrificial oxidizing agents, capture holes to prevent side reactions and enhance the selectivity of the overall process (Ahlawat et al., 2021). In some cases, scavengers serve as redox mediators that facilitate the transfer of electrons or holes between the photocatalyst and the target redox species (Kumar et al., 2021). They act as intermediaries in the electron or hole transfer process, allowing for more controlled and efficient reactions. Scavengers can also stabilize reactive intermediates in the water photo splitting reactions. By capturing transient species, they prevent side reactions and promote the desired pathway, ensuring the efficient production of hydrogen and oxygen (Ramakrishnan et al., 2022).

Overall, scavengers play a pivotal role in water photo splitting reactions by mitigating electron-hole recombination, enhancing charge separation, and facilitating the efficient conversion of water into hydrogen and oxygen. Their careful selection and design are essential for improving the selectivity, efficiency, and overall feasibility of this promising approach to sustainable hydrogen production using solar energy. In this study, Na_2_S/Na_2_SO_3_ mixed salts has been selected to be used scavengers due to its good performance in improving water photo splitting reaction under visible light radiation (Jang et al., 2007).

#### 3.2.1 Mono doping

**FIGURE 5:**
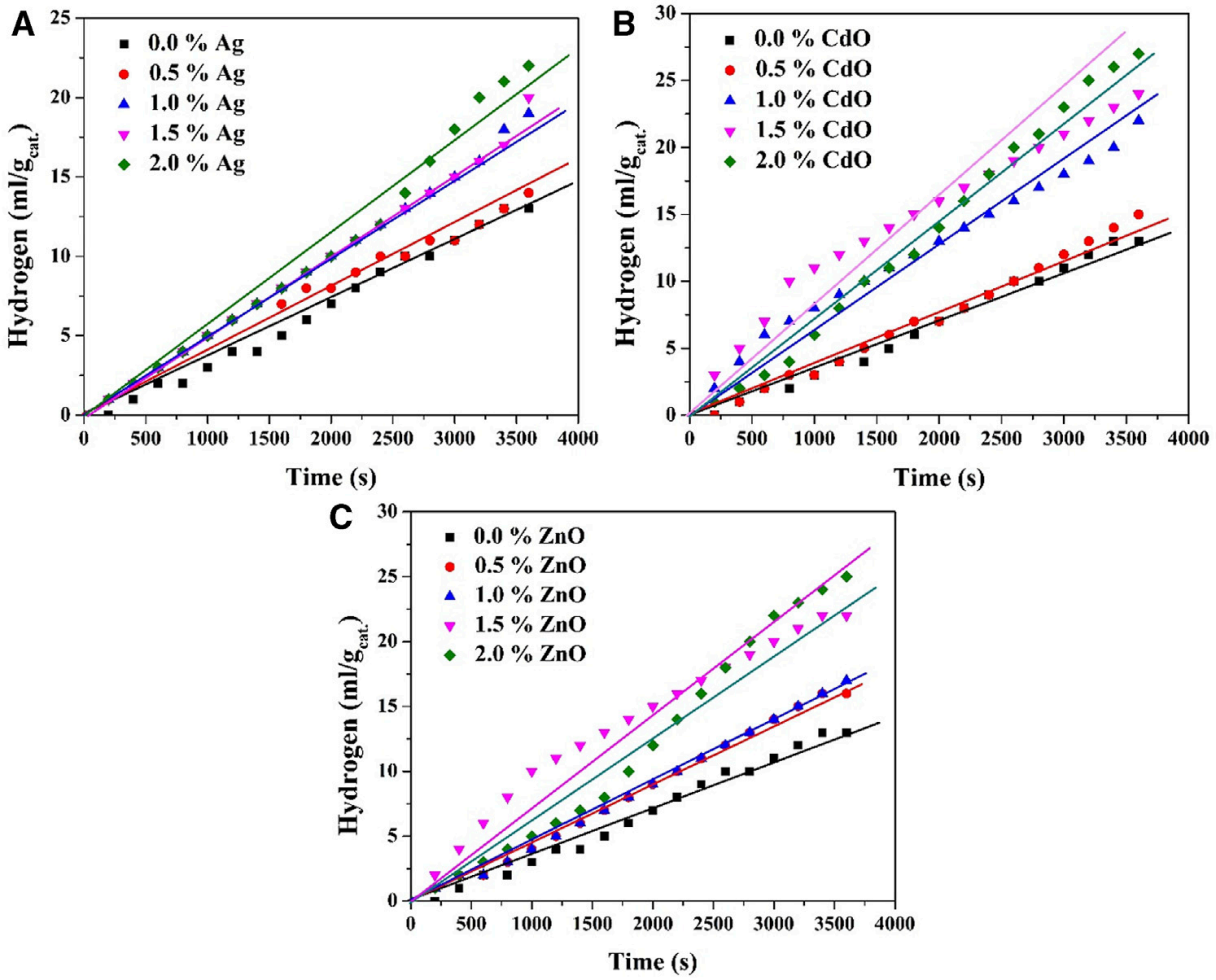
Effect of dopant content on the volume of the generated H_2_ in case of utilizing Ag; **(A)**, CdO; **(B)** and ZnO; **(C)** as photocatalysts when the reaction was performed at 20°C.

The findings presented in Figure 5 concerning the volume of hydrogen evolved from a 0.5 M Na_2_S/Na_2_SO_3_ aqueous solution using Ag, ZnO, and CdO-doped TiO_2_ nanoparticles at various dopant concentrations offer valuable insights into the influence of dopant content on hydrogen evolution. The observations can be discussed as follows: The most prominent trend observed in the results is the positive impact of increasing dopant content on the volume of evolved hydrogen. This phenomenon underscores the pivotal role of dopants in enhancing the photocatalytic activity of TiO_2_-based nanoparticles for water splitting. This outcome aligns with prior research indicating that dopants can extend the lifetime of photogenerated charge carriers, thereby promoting more efficient charge separation and subsequent redox reactions.

In the case of silver nanoparticles as the dopant, a noteworthy observation is the linear proportionality between dopant content and the volume of evolved hydrogen. This suggests that, for silver doping, the enhancement in photocatalytic activity is directly correlated with the quantity of dopant incorporated into the TiO_2_ nanoparticles. The more silver is added, the greater the hydrogen evolution rate, indicating a continuous improvement in catalytic performance.

In contrast, for both zinc oxide and cadmium oxide doping, the results reveal that the optimum dopant concentration for achieving the highest hydrogen evolution rate is approximately 1.5 wt%. Beyond this concentration, there is no significant increase in the volume of evolved hydrogen; instead, the volume of the collected hydrogen is comparatively small. This observation implies that there exists an optimal balance between the dopant content and the photocatalytic efficiency. Exceeding this concentration may lead to a saturation effect, where further doping does not result in additional improvements and may even impede the photocatalytic process. These findings hold significant implications for the design and optimization of photocatalytic systems for water splitting. They suggest that the choice of dopant and its concentration should be carefully considered to achieve the highest hydrogen evolution rate. While silver doping exhibits a linear relationship with dopant content, ZnO and CdO doping exhibit an optimum concentration for maximum efficiency.

Certainly, the observed results in terms of the influence of each dopant (ZnO, CdO, and Ag) can be explained by their respective roles in forming distinct photocatalytic systems within the TiO_2_ nanoparticles. These systems include a type II heterojunction for ZnO, a Z-scheme for CdO, and the creation of a Schottky barrier with noble metals (in this case, Ag).

**FIGURE 6:**
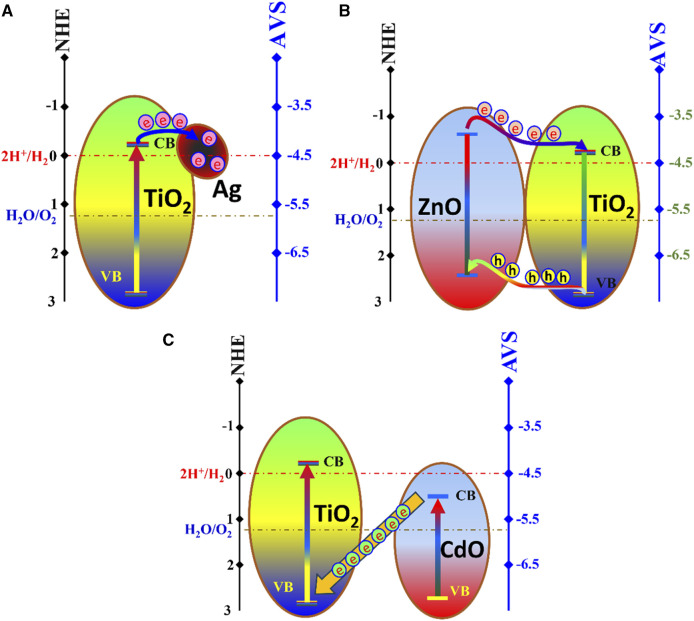
Proposed mechanisms for the investigated photocatalytic systems; Schottky barrier Ag-TiO_2_; **(A)**, type II heterojunction ZnO-TiO_2_; **(B)**, and Z-scheme CdO-TiO_2_; **(C)**.

The Schottky barrier formed by Ag doping influences the hydrogen evolution rate differently compared to the other dopants. Figure 6A illustrates a schematic diagram for the Ag-TiO_2_ photocatalytic system. Briefly, Ag, being a noble metal, creates a Schottky barrier at the Ag-TiO_2_ junction. This barrier prevents the recombination of photogenerated charge carriers by trapping electrons. As a result, the presence of Ag enhances charge separation and facilitates efficient charge transfer to the redox sites, promoting hydrogen evolution. The linear relationship between Ag dopant content and hydrogen evolution rate (Figure 5A) suggests that, in this case, a continuous increase in Ag content results in a proportional increase in photocatalytic activity. This is due to the cumulative effect of more Ag-TiO_2_ junctions, each contributing to enhanced charge separation.

The improvement in the volume of evolved hydrogen upon adding ZnO dopant suggests that ZnO plays a crucial role in enhancing photocatalytic activity. This is indicative of the formation of a type II heterojunction. In the realm of photocatalysis, zinc oxide has risen to prominence as a highly promising contender for environmentally friendly green management systems due to its distinctive attributes. These include a direct and broad band gap within the near-ultraviolet spectrum, robust oxidation capabilities, excellent photocatalytic performance, and a substantial binding energy for free excitons. This remarkable combination of features enables excitonic emission processes to endure even under ambient or elevated temperatures. Accordingly, ZnO can serve individually as a photocatalyst, however, it has a rare application in water photo splitting reaction due to the relatively high gaps between hydrogen ion reduction energy and the metal oxide conduction band (Figure 6B) (Lee et al., 2016). ZnO of TiO_2_ have a close band gap energy (3.37 eV and 3.2 eV, respectively) which concludes simultaneous excitation. In a type II heterojunction, ZnO, with its lower conduction band energy compared to TiO_2_ (as shown in Figure 6B) serves as an efficient electron donor. When illuminated with light, TiO_2_ captures photogenerated electrons from ZnO, preventing their recombination. This prolonged separation of charge carriers enables more electrons to participate in the redox reactions, leading to increased hydrogen evolution. Moreover, holes are accumulated at the valance band of ZnO. Therefore, hydrogen ions reduction takes place at TiO_2_ conduction band, while water oxidation reaction, to produce oxygen gas, could be performed the valance band of ZnO. The trend of increasing hydrogen evolution with higher ZnO content indicates that, up to a certain point, increasing the ZnO content improves charge separation and, hence, photocatalytic activity.

The band gap energy of cadmium oxide (2.3 eV) makes this semiconductor the highest one can absorb the visible light among the three investigated semiconductors in this study. The observation that 1.5 wt% CdO doping leads to the maximum hydrogen evolution rate suggests the formation of a Z-scheme photocatalytic system; Figure 6C. Within a Z-scheme photocatalytic configuration, the undesirable recombination of charge carriers is effectively mitigated by synergizing the photogenerated electrons located in the conduction band (CB) of one semiconductor with the holes situated in the valence band (VB) of another semiconductor. This strategic pairing ensures that the remaining electrons in the CB and holes in the VB possess notably enhanced redox potential, thereby contributing to heightened photocatalytic activity when engaged in photocatalytic reactions. This collaborative mechanism surpasses the photocatalytic performance achievable by individual semiconductors (Yuan et al., 2021). For CdO-TiO_2_ Z-scheme system, CdO absorbs the photon energy, the excited electrons transfer to the valance band of TiO_2_; Figure 6C. Therefore, reduction of hydrogen ions takes place at the conduction band of TiO_2_, while oxygen is produced from water oxidation reaction at the valance band of CdO. The optimum concentration of CdO likely represents the point at which charge separation and transfer are most efficient, maximizing hydrogen evolution.

Overall, the observed differences in the influence of each dopant (ZnO, CdO, and Ag) on hydrogen evolution can be attributed to their respective photocatalytic systems—type II heterojunction, Z-scheme, and Schottky barrier formation. These systems modulate charge separation, electron trapping, and charge transfer differently, ultimately affecting the photocatalytic performance of TiO_2_ nanoparticles in water photo splitting reactions.

**FIGURE 7:**
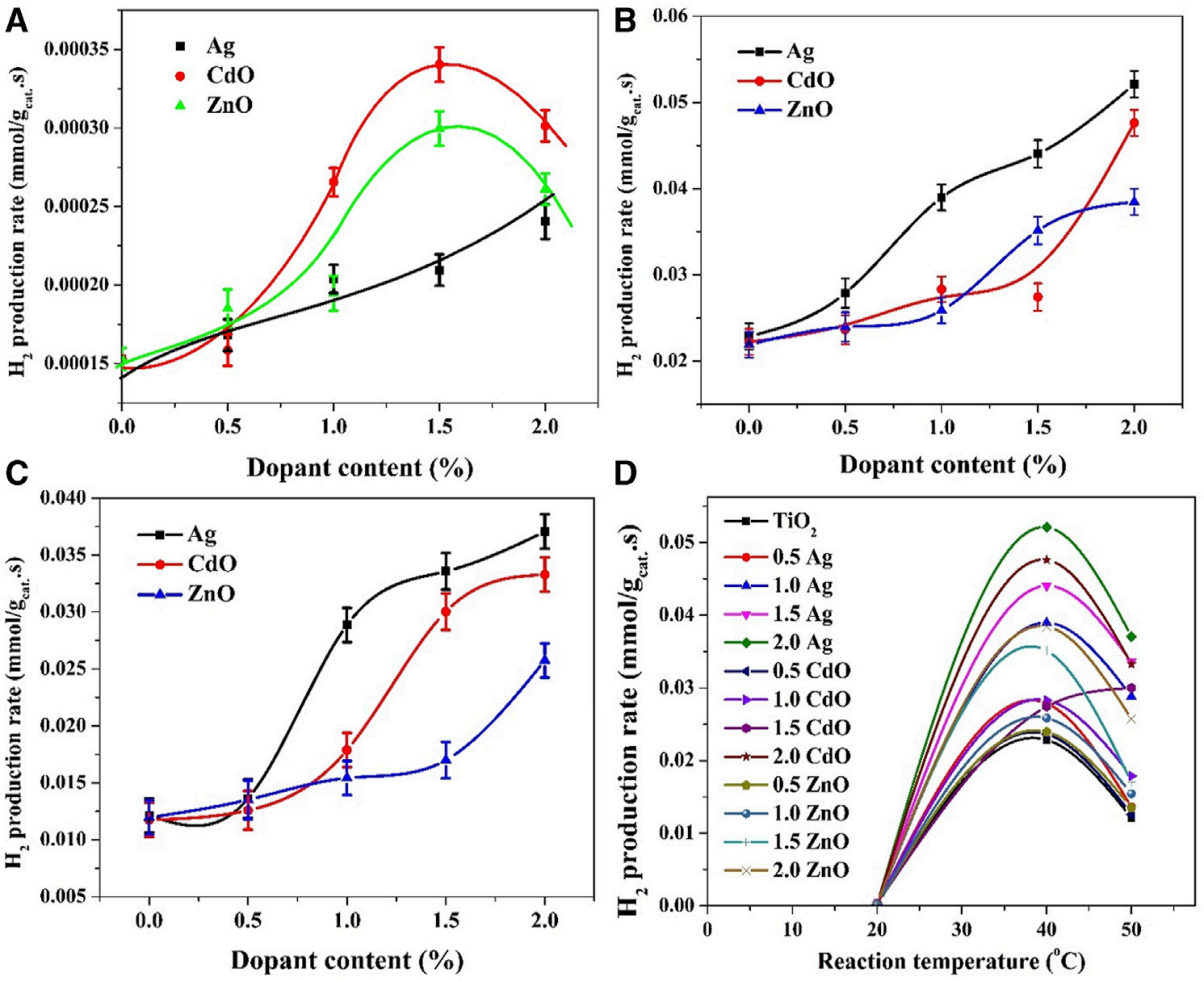
Influence of reaction temperature on the hydrogen production rate; **(A)** at 20°C, **(B)** at 40°C and **(C)** at 50°C. Panel **(D)** summarizes the hydrogen production rates using all formulations at different reaction temperatures.

The observed effects of reaction temperature on the photocatalytic performance of Ag-TiO_2_, ZnO-TiO_2_, and CdO-TiO_2_ formulations, as depicted in Figure 7, reveal intriguing trends in the relationship between dopant content and hydrogen production rate at different reaction temperatures. At low reaction temperature (20°C), the hydrogen production rate exhibits distinct behaviors for the three formulations. For Ag-TiO_2_, there is a linear increase in hydrogen production with increasing silver content. This suggests that at lower temperatures, the presence of Ag dopant significantly enhances charge separation and catalytic efficiency. This finding can be supported by the nature of Ag-TiO_2_ system. In other words, increasing the number of incorporated Ag NPs leads to an increase in the number of electros sinks which results in improving the hydrogen generation reactions. In contrast, both ZnO-TiO_2_ and CdO-TiO_2_ formulations exhibit an optimal dopant content of 1.5 wt% for maximizing hydrogen production. Beyond this concentration, there are diminishing returns. For ZnO-TiO_2_ and CdO-TiO_2_, the presence of an optimal dopant concentration suggests that, at lower temperatures, there may be a balance point where further doping does not significantly contribute to charge separation and, hence, hydrogen production.

The obtained results concerning the effect of increased reaction temperature (40°C and 50°C) on the hydrogen production rates of Ag-TiO_2_, CdO-TiO_2_, and ZnO-TiO_2_ photocatalytic systems reveal interesting trends in the relationship between dopant content and photocatalytic activity. The relationship between dopant content and hydrogen production rate for Ag-TiO_2_ is nearly linear. Increasing the dopant content leads to a consistent increase in the hydrogen production rate. As shown in Figures 7A, C similar trend is observed at 50°C, where increasing the dopant content from 0.5 to 1.0 wt% results in a sharp increase in the gas production rate. Subsequently, a gradual increase in the production rate is noted for 1.5 and 2.0 wt% samples. These trends suggest that at both 40°C and 50°C, Ag dopant plays a significant role in enhancing charge separation and catalytic efficiency. The linear relationship and subsequent gradual increase indicate that the effect of Ag on photocatalytic activity is more pronounced at elevated temperatures. The substantial increase observed at 50°C may be attributed to enhanced reaction kinetics due to the higher thermal energy available to drive the water-splitting reaction.

However, in the case of CdO, at a reaction temperature of 40°C, increasing the dopant content up to 1.5 wt% does have a slight improvement in the hydrogen production rate; from 0.022 to 0.027 mml H_2_/g_cat_.s for pristine and 1.5 wt% samples, respectively. Surprisingly, the production rate increased to 0.0476 mml H_2_/g_cat_.s when the dopant content increased to 2 wt%. In the CdO-TiO_2_ system, the observed behavior at both temperatures suggests that there may be a critical concentration of CdO dopant (around 1.5 wt%) required to initiate a significant improvement in photocatalytic activity. The exact mechanism behind this behavior may involve a complex interplay between charge separation and reaction kinetics, which becomes more pronounced at higher temperatures.

On the other hand, in case of ZnO, at 40°C, a slight increase in the photocatalytic activity was observed up to 1 wt% dopant content, from 0.022 to 0.0258 mml H_2_/g_cat_.s for pristine and 1.0 wt% samples, respectively. Upon increasing the content to 1.5 wt% the production rate was increased to 0.0351 mml H_2_/g_cat_.s with a further small increase to be 0.0384 mml H_2_/g_cat_.s for 2.0 wt% sample.

A little change in the behavior was observed when the temperature was increased to 50°C (Figure 7C). As shown, in the case of Ag, a sharp increase in gas production rate can be seen when the dopant content increased from 0.5 to 1.0 wt%, from 0.0136 to 0.0289 mml H_2_/gcat.s, respectively. Later on, a gradual increase was observed; 0.0336–0.0371 mml H_2_/g_cat_.s for 1.5 and 2.0 wt% samples, respectively. A similar trend could be observed with CdO-TiO_2_ system; however, the threshold is at 1.5 wt% dopant content. Numerically, the observed hydrogen production rates were 0.0118, 0.0126, 0.0179, 0.03 and 0.0333 mml H_2_/g_cat_.s for 0, 0.5, 1.0, 1.5 and 2.0 wt%, respectively. For ZnO dopant, as shown in Figures 7A,C gradual increase was observed up to 1.5 wt% content; from 0.01197 to 0.017 mml H_2_/g_cat_.s for 0 and 1.5 wt% samples, respectively, then a relatively sharp increase in the production rate was observed for the 2.0 wt% sample; 0.0258 mml H_2_/g_cat_.s. Enhancing photocatalytic activity with increasing dopant content at higher temperatures can be attributed to the acceleration of reaction kinetics. Higher temperatures provide more thermal energy to drive the water-splitting reaction, making it less reliant on the dopant’s role in charge separation. Therefore, at higher temperatures, the effect of dopant concentration becomes more pronounced, resulting in a direct proportion between dopant content and hydrogen production rate.

In conclusion, the temperature-dependent trends in the relationship between dopant content and hydrogen production rates for Ag-TiO_2_, CdO-TiO_2_, and ZnO-TiO_2_ photocatalytic systems suggest that the effect of dopants on photocatalytic activity is influenced by both charge separation and reaction kinetics. Elevated temperatures promote enhanced reaction kinetics, leading to more pronounced effects of dopants on photocatalytic activity. Further detailed studies and kinetic modeling are necessary to understand the specific mechanisms underlying these observations.

The summarized results in Figure 7D provide valuable insights into the photocatalytic performance of various formulations at different reaction temperatures, shedding light on the role of dopants and their influence on water splitting reactions. The observation that the water splitting reaction does not follow the Arrhenius equation is a significant finding. The Arrhenius equation typically describes a straightforward exponential relationship between reaction rate and temperature, where higher temperatures lead to faster reaction rates. In this case, the optimum temperature for the water splitting reaction is identified as 40°C, which is contrary to the typical Arrhenius behavior. This deviation from Arrhenius behavior suggests that the reaction kinetics in this system are influenced by factors beyond the simple effect of temperature. The presence of dopants, the specific reaction mechanism, and complex interplay between charge separation and reaction kinetics likely contribute to this non-Arrhenius behavior. Reactions that involve catalysts or inhibitors may not conform to the Arrhenius equation because the presence of these substances can alter the reaction kinetics in a non-linear manner (Barakat et al., 2020; Erfan et al., 2022).

The observation that the formulation with 2.0 wt% silver exhibits the highest photocatalytic activity suggests that creating a Schottky barrier with noble metals has a significant influence on enhancing the photocatalytic properties of TiO_2_-based catalysts for water photo splitting. Schottky barriers formed by noble metals, like silver, effectively trap electrons, preventing their recombination with holes. This enhances charge separation and promotes efficient charge transfer to the redox sites, ultimately leading to higher hydrogen production rates. The superior performance of the 2.0 wt% Ag formulation supports the effectiveness of this approach compared to the other two investigated approaches.

The formulation with 2.0 wt% cadmium oxide is identified as the second-best performer, suggesting that establishing a Z-scheme photocatalytic system also has a positive impact on enhancing the photocatalytic activity of TiO_2_-based catalysts for water photo splitting. In a Z-scheme system, charge carriers are efficiently separated and transferred between different semiconductors. CdO likely plays a crucial role in this charge transfer process, preventing recombination and facilitating enhanced photocatalytic activity. The notable performance of the 2.0 wt% CdO formulation underscores the effectiveness of this approach.

The sequence of the best three formulations, 2.0 wt% Ag, 2.0 wt% CdO, 1.5 wt% Ag, and 1.0 wt% Ag, provides practical insights for optimizing photocatalytic systems for water splitting. These findings suggest that, in the context of TiO_2_-based catalysts for water photo splitting, the creation of Schottky barriers with noble metals and the establishment of Z-scheme photocatalytic systems are promising approaches for enhancing photocatalytic efficiency.

#### 3.2.2 Binary and tertiary doping

**FIGURE 8:**
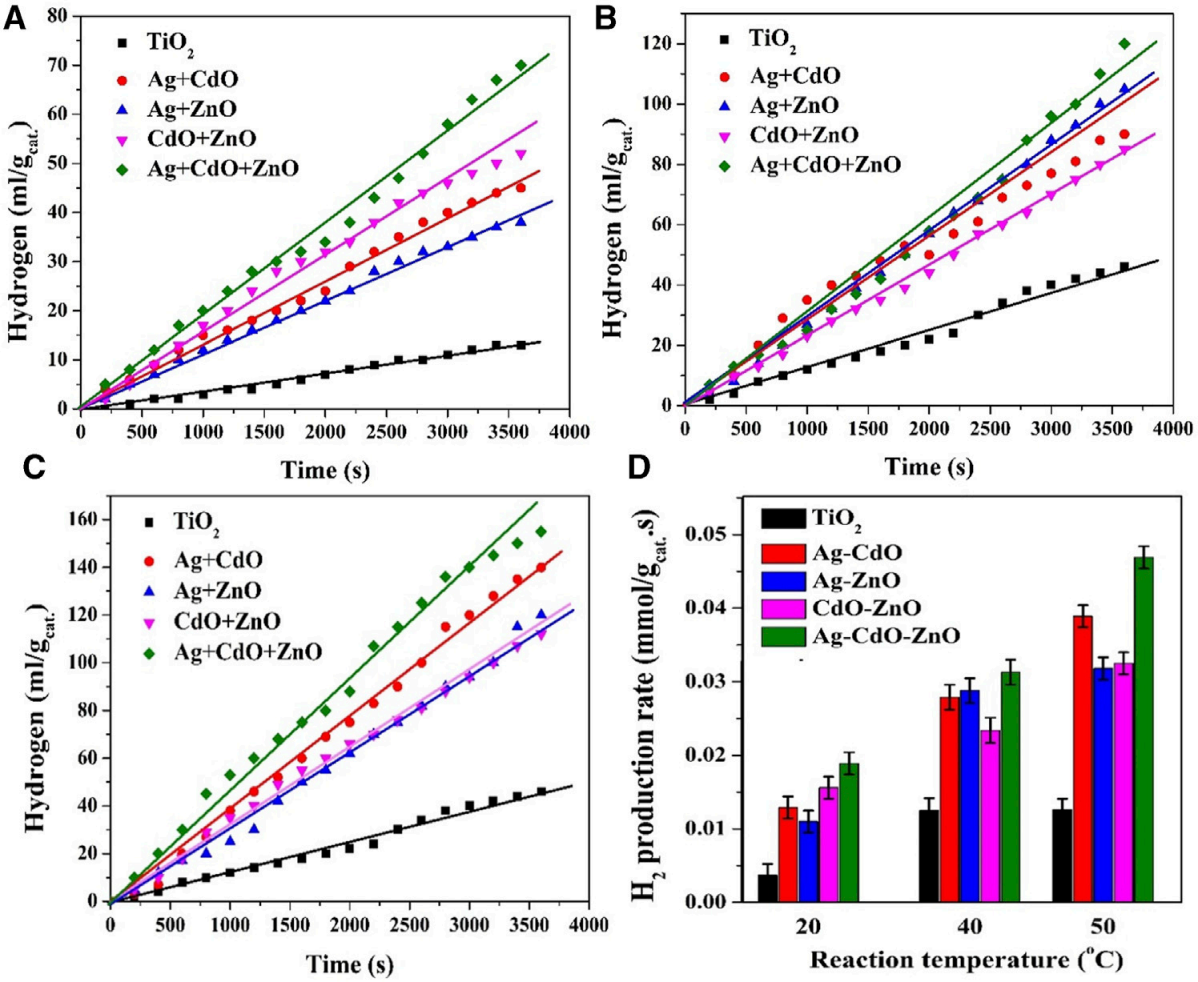
Effect of dopant content on the volume of the generated H_2_ in case of utilizing di and tri doping at various reaction temperatures; 20°C; **(A)**, 40°C; **(B)** and 50°C; **(C)** As photocatalysts when the reaction was performed at 20°C. Panel **(D)** summarizes the hydrogen production rates using all formulations at different reaction temperatures. The dopants content was maintained at 2.0 wt%.

The findings presented in Figure 8 explore the synergetic effect of using di- and tri-dopants in TiO_2_ NPs (2.0 wt% content was used for each dopant) for photocatalytic water splitting at different reaction temperatures (20°C, 40°C, and 50°C). The first important conclusion is that tertiary doping system has the privilege. Typically, the data show that the use of all three dopants (Ag, CdO, and ZnO) simultaneously results in the highest photocatalytic activity at all reaction temperatures. This suggests a beneficial synergy among these dopants in enhancing the water splitting reaction. The synergetic effect of using multiple dopants can be attributed to their complementary roles in charge separation and catalytic promotion. As previously discussed, Ag can create a Schottky barrier, CdO can facilitate Z-scheme systems, and ZnO can contribute to efficient charge separation. Together, they enhance the overall performance of the photocatalyst.

The results conclude also that the impact of dual dopants is dependeny on the reaction temperature. At 20°C, the combination of CdO and ZnO results in the best performance compared to other dual dopant combinations (Ag-CdO and Ag-ZnO). At 40°C and 50°C, the presence of silver in the binary doping system leads to a significant improvement in photocatalytic activity compared to CdO-ZnO binary doping samples. These temperature-dependent effects highlight the complex interplay between dopants and temperature on photocatalytic performance. At lower temperatures (20°C), CdO and ZnO together seem to be more effective, possibly due to their roles in charge separation. Ag may not contribute as significantly at this temperature. At higher temperatures (40°C and 50°C), the elevated thermal energy enhances reaction kinetics. The presence of silver, which can create a Schottky barrier, becomes more influential, leading to improved photocatalytic activity compared to CdO-ZnO combinations.

These results are consistent with the previous observations that demonstrated the effectiveness of Ag in creating Schottky barriers and CdO in forming Z-scheme systems. The synergetic effects of these dopants in the tri-doping configuration likely amplify their individual contributions. In summary, the use of di- and tri-dopants in TiO_2_ NPs for photocatalytic water splitting reveals the importance of considering both the specific dopants and reaction temperature. The findings support the notion that different dopants have varying roles and effectiveness depending on the reaction conditions, which can be harnessed to optimize photocatalytic performance for green hydrogen production.

The interactions and synergistic effects among the dopants in the ternary doping configuration involving Ag, CdO, and ZnO that resulted in enhancing photocatalytic activity are of great interest. The observed enhancements in photocatalytic activity in the ternary doping configuration involving Ag, CdO, and ZnO can be attributed to a combination of interactions and synergistic effects among these dopants. The key interactions and synergies include:

1. Complementary Roles in Charge Separation: Ag, CdO, and ZnO each have distinct roles in improving charge separation within the TiO2 nanoparticles. Ag is known for its ability to create Schottky barriers, which effectively trap electrons, preventing their recombination with holes. CdO has been shown to facilitate Z-scheme systems, where charge carriers are efficiently separated and transferred between different semiconductors. ZnO contributes to efficient charge separation. In the ternary system, these roles are complementary, leading to enhanced charge separation and reduced recombination, which is vital for efficient photocatalysis.
2. Optimization of Reaction Pathways: The synergistic effects also involve optimizing the reaction pathways. CdO and ZnO together appear to be more effective at lower temperatures (20°C), possibly due to their roles in charge separation. Ag, while still beneficial, may not contribute as significantly at this temperature. However, at higher temperatures (40°C and 50°C), the elevated thermal energy enhances reaction kinetics. At these temperatures, the presence of Ag, with its Schottky barrier formation, becomes more influential, leading to improved photocatalytic activity. This temperature-dependent effect underscores the importance of considering reaction conditions in harnessing the synergistic effects of the dopants.
3. Amplification of Individual Contributions: The synergistic effects in the ternary doping configuration likely amplify the individual contributions of Ag, CdO, and ZnO. As previously discussed, each dopant has specific functions that enhance the overall performance of the photocatalyst. When combined, their effects appear to be more than just additive. The presence of all three dopants creates a more complex and interconnected system that improves charge separation, catalytic promotion, and reaction pathways, resulting in higher photocatalytic activity.
4. Tri-Doping Privilege: The results clearly indicate that the tri-doping system, involving all three dopants simultaneously, results in the highest photocatalytic activity at all tested temperatures. This suggests that the synergistic effects of using multiple dopants are more pronounced in the ternary configuration compared to binary or single-dopant systems. The combined influence of Ag, CdO, and ZnO leads to the most significant enhancement in water splitting performance.

The synergetic effects of these dopants in the tri-doping configuration create a highly efficient photocatalytic system. This study highlights the importance of carefully selecting and combining dopants to achieve optimal photocatalytic performance, taking into account the specific roles of dopants and the reaction conditions.

The efficiency of the photoelectrochemical reaction can be evaluated by calculating the quantum efficiency (QE) of water splitting can be calculated from (Maeda, 2011; Qureshi and Takanabe, 2017):

$$\text{QE}=\frac{H_{2}\;\text{gas}\;\text{associated}\;\text{energy}}{\text{Solar}\;\text{beam}\;\text{intensity}}=\frac{r_{H_{2}}\times ∆G_{H_{2}O}^{o}}{I\times A}$$

where, $r_{H_{2}}$ is the reaction rate for water splitting in (mol s^-1^), $∆G_{H_{2}O}^{o}$ is the standard Gibbs free energy for the water-splitting reaction in (J mol^−1^), A is the surface area exposed to solar beams (cm^2^), and I is the intensity of sollar beam at the place of experiment. For Minia City, Egypt, it is about 262 W m^-2^ (Saud et al., 2016). The standard Gibbs free energy for water splitting reaction is 237.1 kJ mol^−1^. Calculations of the QE at $r_{H_{2}}$ = 10.7 mL g_cat_^−1^ min^−1^ showed an efficiency of 36.7%. This figure is increased to around 59.7% in the case of performing the experiment at 50°C, which indicates that one of the slowest steps of the photo-electrochemical reaction is the apparent activation energy of the water-splitting reaction. Such efficiency indicates that the combination of Cd/Zn/Ag over TiO_2_ nanoparticles might function as an auspicious nanocatalyst for the photon-induced water cleavage reaction. Yet, the catalyst stability towards frequent use demands more consideration before proposing it as a possible photocatalyst.

Table 2 provides a valuable comparison of the hydrogen production rates obtained from the proposed tertiary doping system, which includes TiO_2_ NPs doped with Ag, CdO, and ZnO, with those reported for various TiO_2_-based photocatalysts in recent studies. The results demonstrate that the proposed tertiary doping system exhibits very good performance compared to other functional materials. Typically, the hydrogen production rates achieved with the proposed tertiary doping system, as demonstrated in this study, are notably higher compared to other TiO_2_-based photocatalysts. This superior performance can be attributed to the synergistic effects of the three dopants (Ag, CdO, and ZnO) in the ternary configuration. Each dopant plays a specific role in enhancing charge separation and facilitating efficient charge transfer, as elucidated in previous discussions. Ag creates Schottky barriers, CdO supports Z-scheme systems, and ZnO contributes to charge separation. This synergy results in enhanced photocatalytic activity for water splitting.

**TABLE 2:** A comparison of the hydrogen evolution rate for different nanocatalysts.

No	Photocatalyst	Hydrogen production rate, mmol H_2_·g_cat._^−1^ min^−1^, scavenger type	Ref./year
1	Pt/TiO_2_ nanosheet	0.0056	Yu et al. (2010)/2010
2	Graphene modified TiO_2_ nanoparticles	0.0123 (methanol scavenger)	Xiang et al. (2011)/2011
3	ZnO/TiO_2_ mixed oxide	0.0036 (ethanol scavenger)	Pérez-Larios, Lopez et al. (2012)/2012
4	Pt-doped HS-TiO_2_ nanoparticles	0.017 (methanol scavenger)	Zhu et al. (2016)/2016
5	Pt-doped TiO_2_–ZnO	0.0034 (methanol scavenger)	Xie et al. (2017)/2017
6	NiCo_2_S_4_/CdO@CC	0.00125 (not mentioned)	Anwer et al. (2020)/2020
7	Ag-doped TiO_2_	0.391 (methanol, ethanol, or isopropanol scavengers)	Gogoi et al. (2020)/2020
		0.0078 (without scavenger)	
8	ZnCdS/TiO_2_ nanoparticles	0.1406 (Methylene blue scavenger)	Qin et al. (2022)/2022
9	Ag-Fe co-doped TiO_2_ nanoparticles	0.0396 (Methylene blue scavenger)	Sukhadeve et al. (2023)/2023
10	CdTiO_3_ doped TiO_2_ nanoparticles	0.7 (Na_2_S/Na_2_SO_3_ scavenger)	Erfan et al. (2022)/2022
11	(Ag+CdO+ZnO) doped TiO_2_ at 20°C	1.33 (Na_2_S/Na_2_SO_3_ scavenger)	This study
12	(Ag+CdO+ZnO) doped TiO_2_ at 40°C	1.88 (Na_2_S/Na_2_SO_3_ scavenger)	This study
13	(Ag+CdO+ZnO) doped TiO_2_ at 50°C	2.81 (Na_2_S/Na_2_SO_3_ scavenger)	This study

The findings align with the earlier results in this study, which showed that Ag, CdO, and ZnO dopants individually and in combination can enhance charge separation, reduce recombination, and promote efficient photocatalytic reactions. The proposed tertiary doping system harnesses these advantages to achieve superior hydrogen production rates.

In conclusion, the findings presented in Table 2 reaffirm the effectiveness of the proposed tertiary doping system of TiO_2_ NPs with Ag, CdO, and ZnO. This system outperforms many other functional materials and photocatalysts for hydrogen production via water splitting. The study leverages the insights gained from previous results, which highlighted the individual and synergistic roles of these dopants, to provide a comprehensive understanding of why this ternary doping system excels in promoting green hydrogen production. Nevertheless, the stability and recyclability of this photocatalyst must be studied in detail before using it on a large scale.

## 4 Conclusion

The incorporation of Ag, CdO, and ZnO dopants proved highly effective in countering the electron-hole recombination issue, significantly enhancing TiO_2_’s photocatalytic activity for water photo splitting. The dopants’ efficacy and their synergy were found to be temperature-dependent. Notably, Ag emerged as a critical contributor at higher temperatures, leading to substantial gains in photocatalytic performance. For mono doping system, a departure from Arrhenius behavior was observed, revealing an optimal temperature of 40°C for the photocatalytic system. This temperature-dependent behavior sheds light on the intricate dynamics of charge separation and recombination in TiO_2_-based photocatalysts. The ternary doping configuration involving Ag, CdO, and ZnO proved to be the most promising, surpassing many functional materials. In summary, this study showcases the potential of Schottky barriers, Z-scheme systems, and type II heterojunctions, when paired with specific dopants, to overcome the electron-hole recombination challenge in TiO_2_-based photocatalysis. This research not only enhances our understanding of photocatalysis but also offers a promising pathway for sustainable energy applications. To build upon the insights gained in this study, future research endeavors can focus on investigating alternative ternary doping configurations with different dopants to further optimize photocatalytic performance and uncover new synergistic effects. Assessing the scalability and practicality of the proposed ternary doping system for real-world applications, such as large-scale hydrogen production or wastewater treatment, is also recommended.

## Data Availability

The original contributions presented in the study are included in the article/Supplementary Material, further inquiries can be directed to the corresponding authors.
