# Supplementary material for: Synergistic doping with Ag, CdO, and ZnO to overcome electron-hole recombination in TiO2 photocatalysis for effective water photo splitting reaction
Source: Front Chem. 2023 Nov 6;11:1301172. doi: 10.3389/fchem.2023.1301172 (PMC10661415; doi:10.3389/fchem.2023.1301172)
Supplement: Supplementary file 1 [file DataSheet1.PDF]

Synergistic Doping with Ag, CdO, and ZnO to Overcome Electron-Hole Recombination in TiO_2 Photocatalysis for Effective Water Photo Splitting Reaction

Nehal A. Erfan[1], Mohamed S. Mahmoud[1, 2], Hak Yong Kim,[3,4,*] Nasser A. M. Barakat[1,*]

[1]Chemical Engineering Department, Minia University, El-Minia, 61516, Egypt

[2]University of Technology and Applied Sciences, Department of Engineering, Suhar, 311, Sultanate of Oman

[3]Department of Nano Convergence Engineering, Jeonbuk National University, Jeonju 54896, South Korea

[4]Department of Organic Materials and Fiber Engineering, Jeonbuk National University, Jeonju 54896, South Korea

Corresponding authors:

Nasser A. M. Barakat,

Tel: +20862348005, Fax: +20862364420

E-mail: *nasbarakat@mu.edu.eg*

Hak Yong Kim

E-mail: **khy@jbnu.ac.kr**

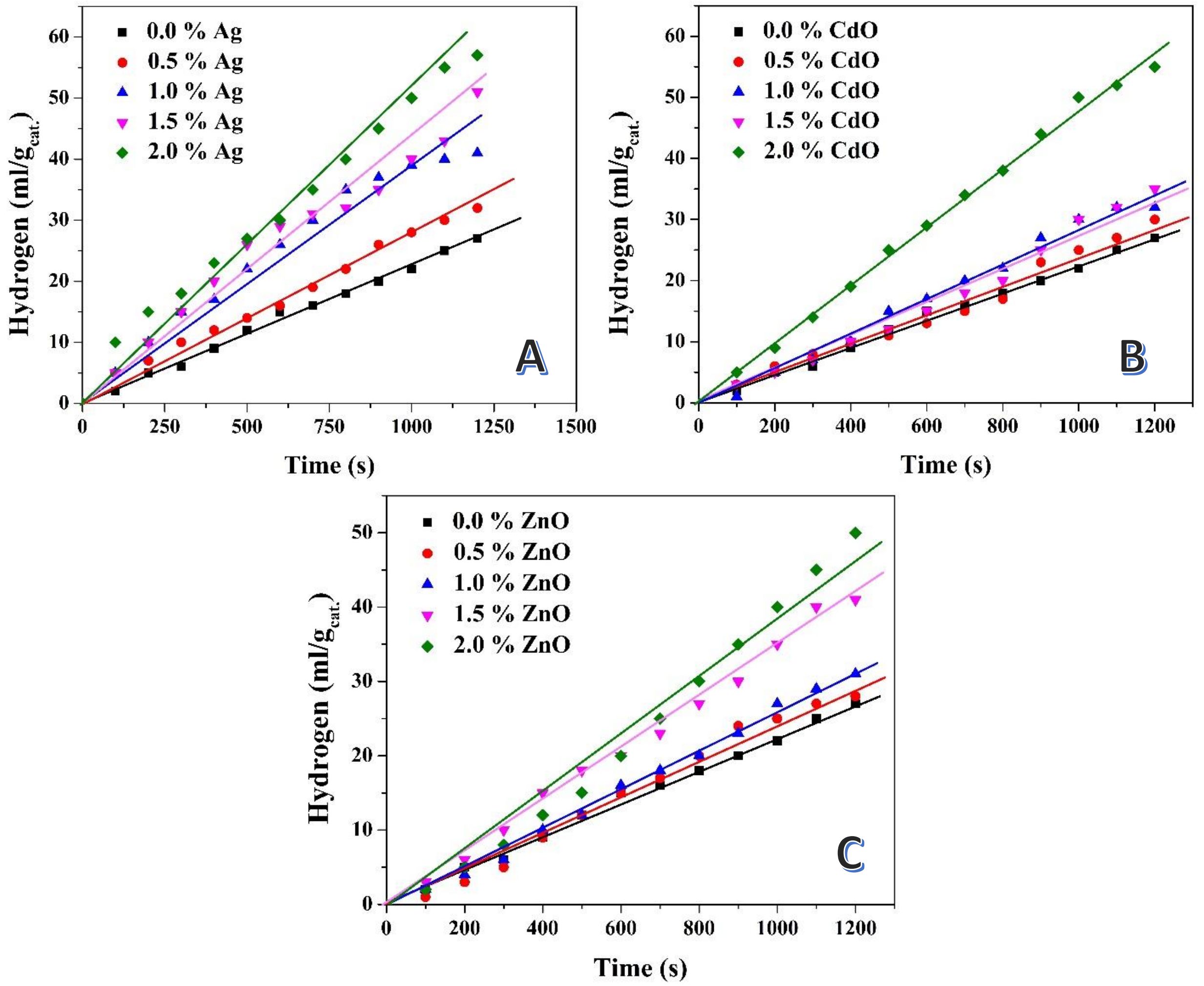

Figure S1. Effect of dopant content on the volume of the generated H_2 in case of utilizing Ag; (A), CdO; (B) and ZnO; (C) as photocatalysts when the reaction was performed at 40 ^oC.

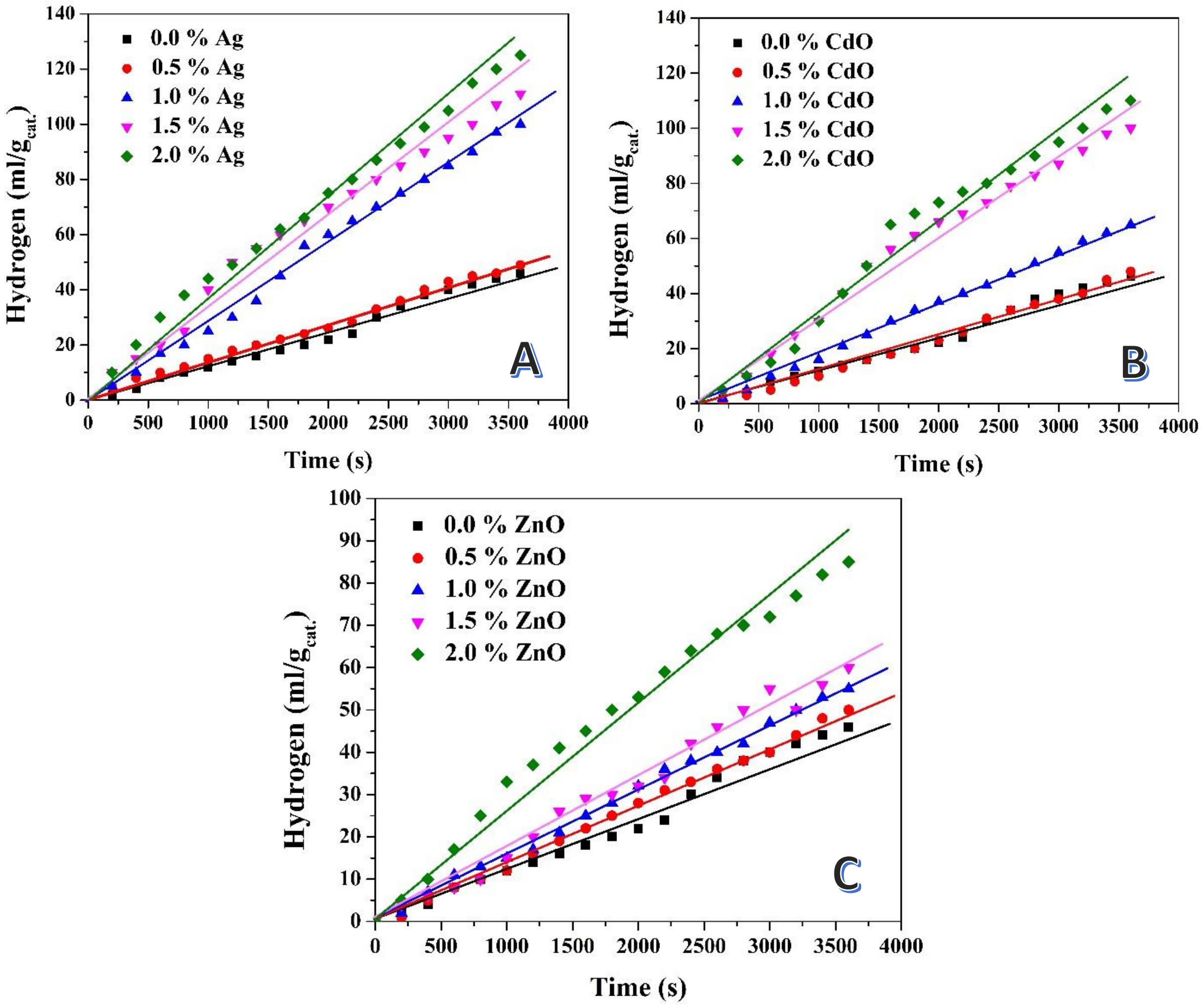

Figure S2. Effect of dopant content on the volume of the generated H_2 in case of utilizing Ag; (A), CdO; (B) and ZnO; (C) as photocatalysts when the reaction was performed at 50 ^{o}C.
